# FAK regulates leptin-induced angiogenesis and vasculogenic mimicry in breast cancer

**DOI:** 10.1007/s12032-026-03370-y

**Published:** 2026-08-29

**Authors:** Ana K. Herrera-Vargas, Ricardo Jaime-Cruz, Alejandra Rodríguez-Leviz, Miguel A. Mendoza-Catalán, Monserrat Olea-Flores, Laura Villavicencio-Guzmán, Marcela Salazar-García, Carlos C. Patiño-Morales, Napoleón Navarro-Tito

**Affiliations:** 1 Laboratorio de Biología Celular del Cáncer, Universidad Autónoma de Guerrero. Chilpancingo, Guerrero, México; 2 Laboratorio de Investigación en Biología del Desarrollo y T.E. Hospital Infantil de México Federico Gómez, Ciudad de México, México; 3 Laboratorio de Investigación en Bioactivos y Cáncer, Universidad Autónoma de Guerrero. Chilpancingo, Guerrero, Mexico; 4 Department of Biochemistry and Molecular Biotechnology, University of Massachusetts Chan Medical School, Worcester, MA 01605 USA; 5 Laboratorio de Biología Celular, Universidad Autónoma Metropolitana - Cuajimalpa, Ciudad de México, México

**Keywords:** Leptin, Angiogenesis, Vasculogenic mimicry, FAK, Breast cancer

## Abstract

Leptin, an adipokine primarily secreted by adipose tissue, has been implicated in tumor progression by regulating angiogenesis. Leptin also plays a key role in tumor vascularization by promoting vasculogenic mimicry (VM). During tumor progression, leptin activates several signaling pathways, including the focal adhesion kinase (FAK) pathway. This study aimed to determine whether leptin promotes and regulates angiogenesis and VM through the FAK pathway. We used the chick chorioallantoic membrane (CAM) as a model to evaluate blood vessel formation and to induce tumors to assess angiogenic expression. Matrigel-based cell cultures were used to analyze the formation of tubular structures characteristic of VM. The expression of specific markers was then evaluated for each process. These models were employed with or without the inhibitor PF-573,228. Our results showed that leptin enhances the sprouting, branching, and vasodilation of blood vessels via FAK signaling by increasing the expression of vascular endothelial growth factor (VEGF) and N-cadherin in tumors derived from MCF-7 and MDA-MB-231 cells. Furthermore, leptin stimulation promoted tubular-type VM in MCF-7 cells and matrix-type VM in MDA-MB-231 cells; the former was dependent on FAK signaling. Lastly, leptin increased the levels of several proteins associated with angiogenesis and VM, including TIE-1, MMP-9, VE-cadherin, ANG-2, VEGF, and VEGFR1. In conclusion, leptin promotes tumor vascularization in breast cancer through angiogenesis and VM, in a manner dependent on FAK signaling

## Introduction

 Breast cancer (BC) is the most common cancer in women, representing the leading cause of cancer-related death and accounting for 16% of all female malignancies [1]. BC subtypes differ in prognosis and therapeutic targets, including luminal A, the most prevalent among patients, and triple-negative breast cancer, which exhibits aggressive behavior, chemoresistance, and poor prognosis [2]. Poor prognosis is associated with alterations in several hallmark processes, including induction of and access to the vasculature [3].

Angiogenesis is defined as the formation of new blood vessels from existing vasculature [4]. Vascular endothelial growth factor (VEGF) and its receptors, VEGFR1 and VEGFR2, are among the key regulators of tumor angiogenesis and are considered the principal stimulators [5]. They orchestrate cellular proliferation, transformation, migration, apoptosis, and vascularization [6, 7]. Similarly, angiopoietins (ANGs) and their receptors TIE-1 and TIE-2 are essential for endothelial cell survival, vascular stability, and maturation [8].

In addition to angiogenesis, alternative mechanisms of tumor vascularization have been described, including vasculogenic mimicry (VM) [9]. VM is an alternative neovascularization process adopted by tumors to evade antiangiogenic therapies and adapt to a hypoxic microenvironment [10]. During VM, tumor cells undergo transdifferentiation toward an endothelial-like phenotype, facilitating the formation of fluid-conducting tubular networks that supply oxygen and nutrients [11, 12].

VE-cadherin has been identified as the key molecular mediator of VM. VE-cadherin directs the recruitment of EphA2 to intercellular junctions, forming the characteristic tubular channels of VM [13]. EphA2 activation subsequently triggers the PI3K and ERK1/2 signaling pathways, which support tumor cell survival, proliferation, and migration [14]. Matrix metalloproteinases (MMPs) facilitate VM by degrading the extracellular matrix and enabling tubular channel formation [15]. Specifically, MMP-2 and MMP-9 are overexpressed in VM-positive tumors [16]. In addition, the tumor microenvironment plays a crucial role in vascularization.

Cancer-associated adipocytes, the predominant stromal cells in the breast tumor microenvironment, secrete chemokines such as leptin, which can enhance tumor cell proliferation, migration, invasion, and angiogenesis [17, 18]. Focal adhesion kinase (FAK) is among the signaling mediators of leptin’s effects. Leptin activates FAK in MDA-MB-231 and MCF-7 cells, promoting secretion of MMP-2 and MMP-9 and thereby driving breast cancer cell migration and invasion [19]. Similarly, in non-tumorigenic MCF10A cells, leptin‐induced FAK activation leads to epithelial-to-mesenchymal transdifferentiation, MMP-2/−9 secretion, invadopodia formation, and increased invasive capacity [20].

Leptin has been described to participate in tumor vascularization; however, the mechanisms through which it regulates angiogenesis and VM remain poorly understood. Although FAK has been widely implicated in angiogenesis and tumor progression, its role as a regulator of leptin-mediated effects remains poorly understood, underscoring a significant gap in the current literature. Based on the above, we hypothesize that leptin regulates angiogenesis and VM through the noncanonical FAK pathway in breast cancer cells.

## Materials and methods

### Reagents

Human leptin (L4146-1MG, Sigma-Aldrich); FAK inhibitor PF-573,228 (869288-64-2, Sigma-Aldrich); Primary antibodies: anti-p-FAK-pY397 (AP0302), anti-VEGF (A12303), anti-TIE-1 (A15104), anti-ANG-2 (A0698), anti-VEGFR1 (A19132), anti-MMP-9 (A0289), anti-VE cadherin (A0734) were obtained from ABclonal; anti-N-cadherin (sc-8424), anti-VEGF (Sc-7296) obtained from Santa Cruz. Secondary antibodies: anti-mouse (Sc-2368), anti-goat (Sc-2348) were obtained from Santa Cruz, anti-rabbit (AS014, ABclonal); Matrigel^®^ Matrix (354234, Corning); Fertilized chicken embryos (*Gallus gallus*; ALPES S.A. de C.V.); Fetal bovine serum (Byproductos, SA de CV); Antibiotic–antimycotic solution (penicillin, streptomycin, amphotericin B; A5955, Sigma-Aldrich).

### Cell culture

The breast cancer cell lines MCF-7 (ATCC^®^ HTB-22) and MDA-MB-231 (ATCC^®^ HTB-26) were maintained in DMEM/F12 medium (Sigma-Aldrich) supplemented with 5% fetal bovine serum and 1% antibiotic–antimycotic solution. Cultures were incubated at 37 °C in a humidified atmosphere containing 5% CO₂. For experimental treatments, cells were serum-starved for 24 h before the addition of PF-573,228 and/or leptin.

### Cell stimulation

MCF-7 and MDA-MB-231 cells were plated in 60 mm dishes containing 3 mL of DMEM/F12. At ~ 80% confluence, cultures were rinsed with 1× PBS and treated with PF-573,228 (5 µM) for 2 h and/or leptin (200 or 100 ng/mL) for 24 h. All experiments were conducted using three independent biological replicates.

### Western blot

After treatment, the cells were lysed in RIPA buffer (50 mM HEPES, pH 7.4; 150 mM NaCl; 1 mM EGTA; 1 mM sodium orthovanadate; 100 mM NaF; 10 mM sodium pyrophosphate; 10% glycerol; 1% Triton X-100; 1% sodium deoxycholate; 1.5 mM MgCl₂; 0.1% SDS) containing 1 mM PMSF (Sigma-Aldrich). Total protein (30 µg) was separated on 10% SDS-PAGE gels and transferred to nitrocellulose membranes (Bio-Rad). Subsequently, membranes were blocked with 3% BSA in TBS-T (0.05% Tween 20) for 2 h at room temperature, then incubated overnight at 4 °C with primary antibodies against VEGF, ANG-2, VEGFR1, TIE-1, VE-cadherin, MMP-9 and p-FAK (pY397) (all at 1:1000). After washing, HRP-conjugated secondary antibodies (1:5000) were applied for 2 h at room temperature. Detection was performed using enhanced chemiluminescence (Bio-Rad), and band intensities were quantified by densitometry with ImageJ (v1.52p). Western blot experiments were performed using three independent biological replicates.

### CAM assay

Pathogen-free, fertilized chicken eggs (*Gallus gallus*) were incubated at 37 °C in a humidified, rotating chamber. On day 7, a 1 cm² opening was made over the embryo, and sterile filters soaked in solutions with varying concentrations of leptin (50, 100, 200, and 400 ng/mL), with or without 5 µM of PF-573,228, were placed on the CAM. On day 12, the embryos were euthanized, and the filters were retrieved and imaged under a stereomicroscope [21]. Representative images were captured digitally (ZEISS Axio Zoom V16, 30x) for morphometric analysis using ImageJ (v1.52p). Treatments were evaluated in three independent biological replicates (*n* = 3 embryos per condition). From each replicate image, total sprouting and branching were quantified, and three measurements of vessel diameter were obtained per replicate.

### Tumor induction on the CAM

Fertilized eggs were incubated at 37 °C with 90% humidity. On day 7, a 1 cm² window was cut to expose the CAM, and xenografts were implanted by depositing 30 µL of Matrigel containing MCF-7 or MDA-MB-231 cells (3 × 10^6^ cells). Tumor formation was monitored for four days. On day 11, tumors were treated with leptin (500 ng/mL) for 48 h, then excised, fixed in 4% paraformaldehyde, dehydrated with a series of alcohols of increasing concentration (from 30% to absolute), rinsed in xylene, and embedded in paraffin. Three tumors were induced per treatment condition (*n* = 3), with one tumor generated per embryo.

### Immunofluorescence

Paraffin-embedded xenografts were sectioned coronally at 5 μm (Leica microtome) and mounted on slides. Sections were rehydrated, and antigen retrieval was performed in citrate buffer (BioGenex) at 15 psi in an autoclave for 5 min. Samples were incubated overnight at 4 °C with anti-VEGF (1:200) and anti-N-cadherin (1:250), followed by fluorescent secondary antibodies (1:200) for 4 h at room temperature. Nuclei were stained with Redox (1:150), and images were acquired on a Carl Zeiss LSM 780 NLO confocal microscope at 40× magnification (Zen, 2010). Protein expression was evaluated in three independent biological replicates (*n* = 3 tumors per treatment condition). For each replicate, three representative images were analyzed. Fluorescence intensity was quantified using ImageJ software (v1.52p).

### Histological analysis of blood vessels in xenografts

Xenograft tissues were fixed in 4% paraformaldehyde, embedded in paraffin, sectioned at 5 μm, placed on slides, and heated overnight at 40 °C under a histology oven. Then, the sections were cleared from paraffin after three changes in xylene, 5 min each. The tissue was rehydrated through a series of washes with decreasing alcohol concentrations (100–96%). The rehydration step was completed by rinsing in distilled water for at least 5 min. The slides were stained with Mayer’s hematoxylin solution for 3 min, followed by two quick washes (tap water and distilled water, respectively). The tissues were then immersed in acidulated alcohol (3 s) and ammonia water (3 s). Then, the same slides were stained with eosin for 30 s. The tissue was dehydrated through another series of washing, this time with increasing alcohol concentration (96–100%, 3 s). The procedure concluded with two changes in xylol (3 s each). Histological images were acquired using an Aperio ScanScope at 20× magnification. Three tumors per treatment condition were analyzed (*n* = 3). For vessel quantification, two sections per tumor were analyzed to account for variability due to sectioning. The number of vessels was quantified in each section. For vessel diameter analysis, six measurements per tumor were performed for MCF-7 xenografts, whereas ten measurements per tumor were performed for MDA-MB-231 xenografts due to their higher vessel density.

### Vasculogenic mimicry assay

To assess VM, 50 µL of Matrigel (diluted 1:5 in DMEM with 1% FBS) was added per well of a 96-well plate and allowed to solidify at 37 °C. MCF-7 or MDA-MB-231 cells (2.5 × 10⁴ cells/well) were seeded in DMEM with 1% FBS containing leptin (50, 100, 200, and 400 ng/mL) with or without PF-573,228 for 48 h [22, 23]. Three-dimensional tumor cultures were stained with Periodic Acid-Schiff (PAS) according to standard procedures. Briefly, cells were fixed for 30 min at room temperature in 4% formaldehyde. Cells were then pretreated for 5 min with 5% periodic acid solution, followed by a 1-minute wash in distilled water. Cells were then incubated with Schiff’s reagent for 15 min at room temperature, and the plates were subsequently washed for 15 min in tap water with gentle agitation [24]. After staining, each well was analyzed directly under an inverted phase-contrast microscope (Leica Microsystems DMi8 A). Images of the channels were captured in different fields at 10× magnification. One representative image was selected for each treatment, and the number of tubes formed was quantified using ImageJ software (v1.52p); the number of channels formed was then averaged.

As the main criterion, only channels with a clearly defined lumen and a circular or ovoid shape were considered for quantification. Structures that did not meet this criterion were excluded to avoid including simple cellular alignments without a defined lumen or incomplete channels [25]. All experiments were performed in triplicate.

### Statistical analysis

Data are presented as mean ± SEM. Statistical analyses were performed using one-way ANOVA followed by Student’s t-test, Dunnett’s test, or Newman–Keuls multiple-comparison test, as appropriate, using GraphPad Prism v5.0. A p-value < 0.05 was considered statistically significant.

## Results

### Leptin promotes angiogenic development

**Fig. 1:**
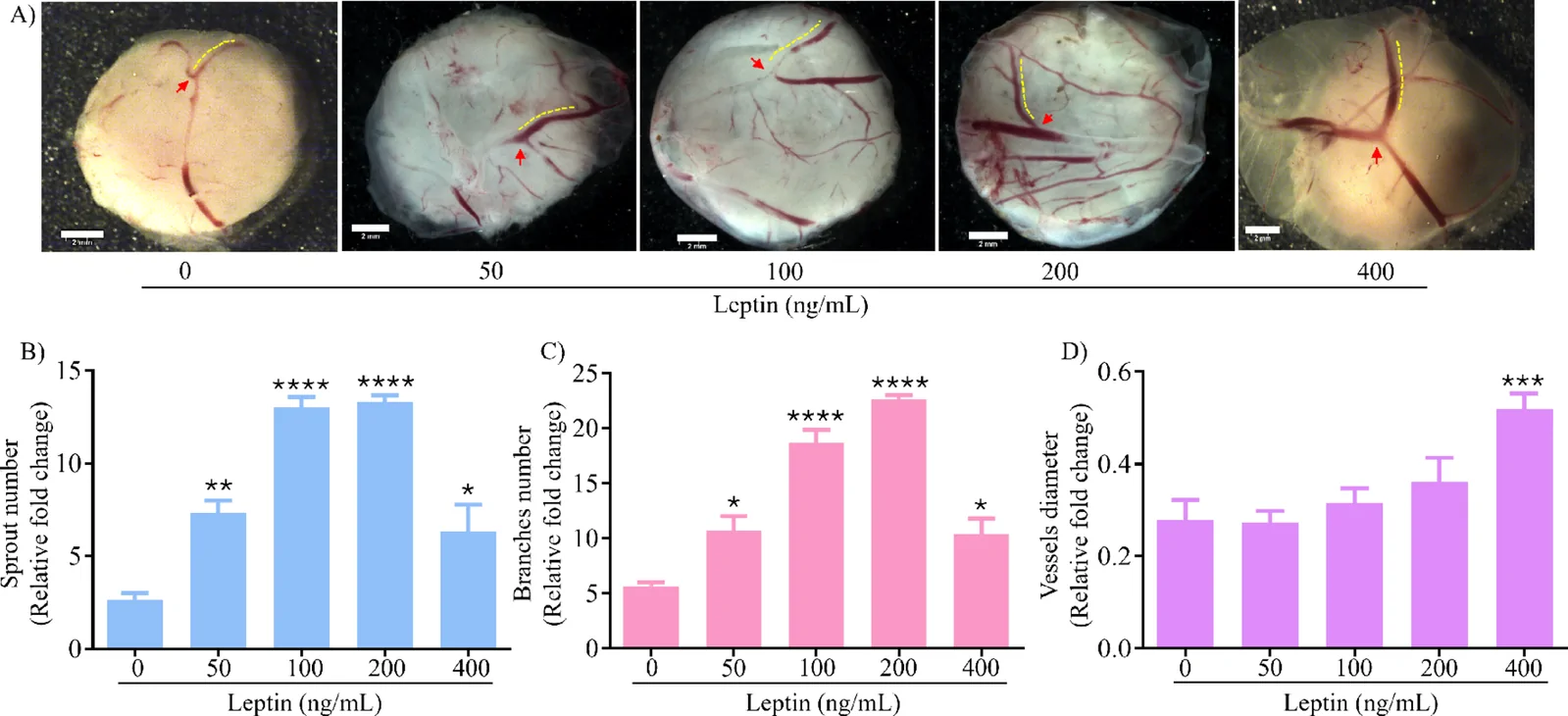
Leptin promotes angiogenesis. (**A**) Representative images of the CAM assay after 120 h of leptin treatment. Red arrows indicate capillary sprouts; yellow dashed lines highlight vessel branching. (**B**) Representative graph of capillary sprout quantification. (**C**) Representative graph of vessel branching quantification. (**D**) Representative graph of capillary diameter analysis. Data are presented as mean ± SEM. One-way ANOVA and Dunnett’s test: *p < 0.05, ** *p* < 0.01, *** *p* < 0.001, **** *p* < 0.0001. Scale bar = 2 mm

Leptin has been identified as a potent angiogenic factor; however, conflicting findings indicate that its specific role in the regulation of angiogenesis remains a matter of debate [26]. To evaluate the angiogenic effect of leptin, we used the CAM model, as previously described, which provides a highly vascularized and accessible system for experimental manipulation [21]. Our results showed that leptin promoted capillary sprouting at concentrations as low as 50 ng/mL (7.33 ± 0.67, *p* < 0.0062), with maximal activity observed at 200 ng/mL (13.33 ± 0.33 vs. 2.67 ± 0.33, *p* < 0.0001, respectively, *n* = 3; Fig. 1A and B). In addition, leptin increased vessel branching in a dose-dependent manner from 50 ng/mL (10.67 ± 1.33, *p* < 0.0234) to 200 ng/mL (22.67 ± 0.33 vs. 5.67 ± 0.33, *p* < 0.0001, respectively, *n* = 3; Fig. 1A and C). These findings are consistent with classical angiogenic responses characterized by enhanced neovascular formation and increased vascular complexity. Interestingly, only treatment with 400 ng/mL leptin produced a significant increase in capillary diameter (0.52 ± 0.03 vs. 0.28 ± 0.04, respectively, *n* = 3 (9 measurements), *p* < 0.0003 Fig. 1A and D). This increase in vascular caliber suggests vascular remodeling rather than de novo angiogenesis. Therefore, while low leptin concentrations promote classical angiogenic responses, higher concentrations appear to induce structural modifications in blood vessels. Taken together, these data suggest that leptin may enhance tumor perfusion and, consequently, the supply of oxygen and nutrients. In addition, it may enhance vascular permeability and facilitate cellular extravasation through vascular remodeling.

### Leptin enhances pro-angiogenic factor expression and vascular development in breast cancer tumors

**Fig. 2:**
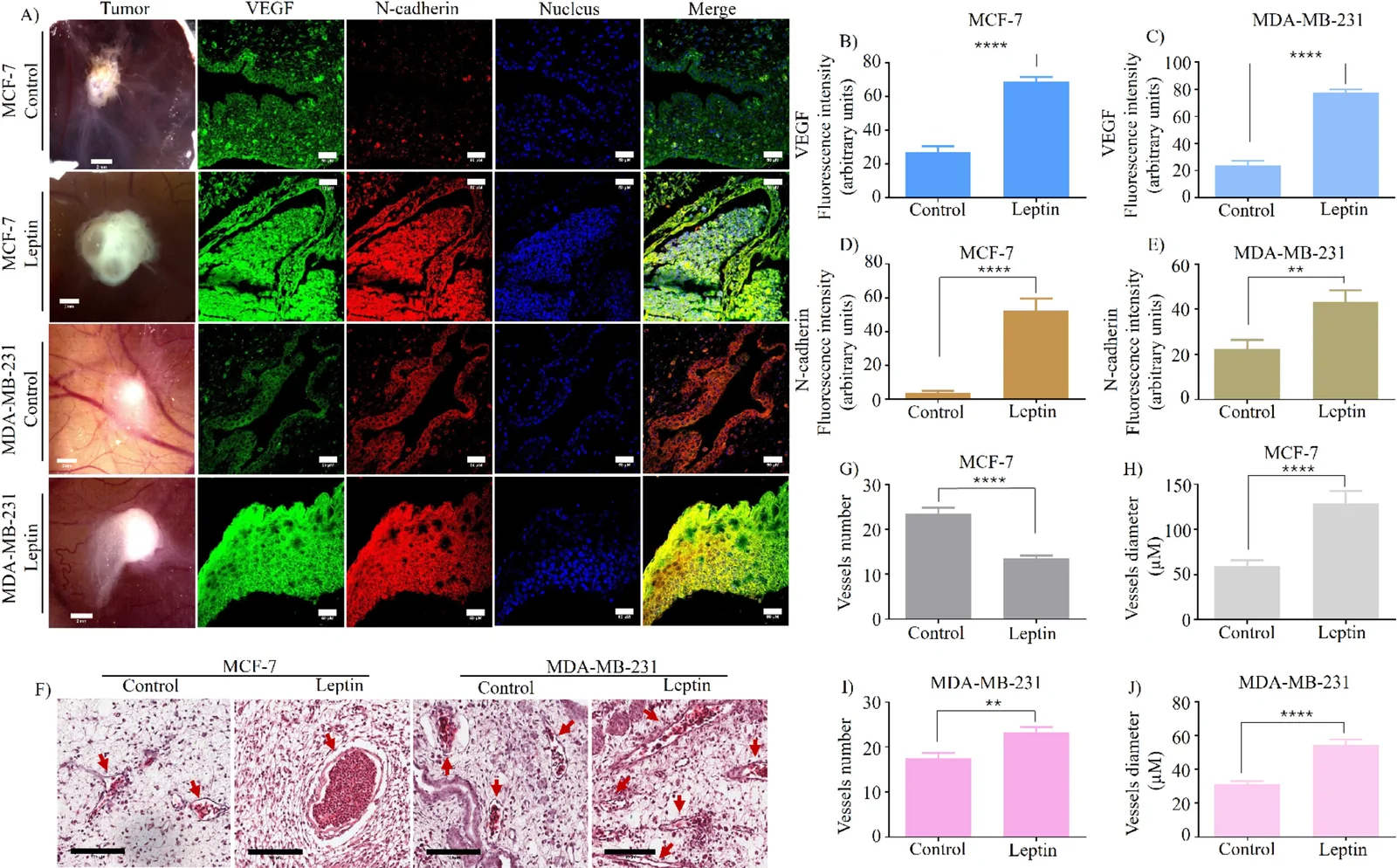
Leptin induces pro-angiogenic protein expression and vascular development in luminal A and triple-negative breast cancer tumors. **A.** Representative images of immunofluorescence of MCF-7 and MDA-MB-231-derived tumors treated with leptin for 48 h (scale bar in tumors = 2 mm; scale bar in immunofluorescence = 50 μm). Green: VEGF, Red: N-cadherin, Blue: nuclei. **B**–**C.** Representative graphs of the quantification of VEGF expression in MCF-7 and MDA-MB-231 tumors. **D**–**E.** Quantification of N-cadherin levels in MCF-7 and MDA-MB-231 tumors. F) H&E-stained tumor sections from MCF-7 and MDA-MB-231 tumors treated with leptin for 48 h, scale bar = 100 μm. Red arrows indicate blood vessels. G–H) Analysis of vessel number and diameter in MCF-7 tumors. **I**–**J.** Analysis of vessel number and diameter in MDA-MB-231 tumors. Data are presented as mean ± SEM. Statistical significance was determined using Student’s t-test: *p < 0.05, ** *p* < 0.01, *** *p* < 0.001, **** *p* < 0.0001

The tumor microenvironment plays an important role in processes associated with tumor development and progression, including angiogenesis, invasion, and metastasis [27]. MCF-7 and MDA-MB-231 cells were implanted on the CAM, and tumors were stimulated with leptin (500 ng/mL) to evaluate the relationship between leptin, tumor cells, and the vascular microenvironment. Leptin stimulation significantly increased the expression of VEGF and N-cadherin in both tumor types. VEGF levels increased from 68.65 ± 2.77, vs. 27.01 ± 3.65, *p* < 0.0001 in MCF-7 and 77.33 ± 2.61 vs. 23.88 ± 3.35, *p* < 0.0001 in MDA-MB-231, while N-cadherin expression increased 52.20 ± 7.22 vs. 3.90 ± 0.98, *p* < 0.0001 and from 43.41 ± 5.03 vs. 22.59 ± 3.80, *p* < 0.0045 respectively (*n* = 3 tumors, 9 measurements Fig. 2A–E).

Histological analysis revealed distinct vascular responses between the two tumor models. Leptin-treated MCF-7 tumors exhibited fewer blood vessels (13.67 ± 0.56 vs. 23.50 ± 1.38, *p* < 0.0001, *n* = 3 tumors, 6 measurements); however, leptin significantly increased vessel diameter (128.7 ± 13.77 vs. 59.75 ± 5.94, *p* < 0.0001, *n* = 3 tumors, 18 measurements, Fig. 2F-H), suggesting structural vascular changes consistent with vascular remodeling. In contrast, leptin treatment in MDA-MB-231 tumors promoted both an increase in vessel number (23.33 ± 1.12 vs. 17.50 ± 1.23, *p* < 0.0056, *n* = 3 tumors, 6 measurements, Fig. 2I) and vessel diameter (54.34 ± 3.32 vs. 31.31 ± 1.65, *p* < 0.0001, *n* = 3 tumors, 30 measurements, Fig. 2F, I, J). These differential vascular responses are consistent with the vascular alterations observed in the CAM model in the absence of tumor cells and may reflect the distinct intrinsic angiogenic potential of these breast cancer subtypes. While the luminal subtype reorganizes the vascular network, which may reflect slower and more organized tumor growth, triple-negative tumors increase vascular density, potentially promoting rapid tumor growth, a higher risk of metastasis, and greater dependence on angiogenic signaling.

### Leptin promotes vasculogenic mimicry in breast cancer cells

Although leptin is known to influence tumor vascularization, its role in VM remains poorly understood. In this study, MCF-7 and MDA-MB-231 cells were seeded on Matrigel and treated with different concentrations of leptin (50, 100, 200, and 400 ng/mL) for 48 h.

**Fig. 3:**
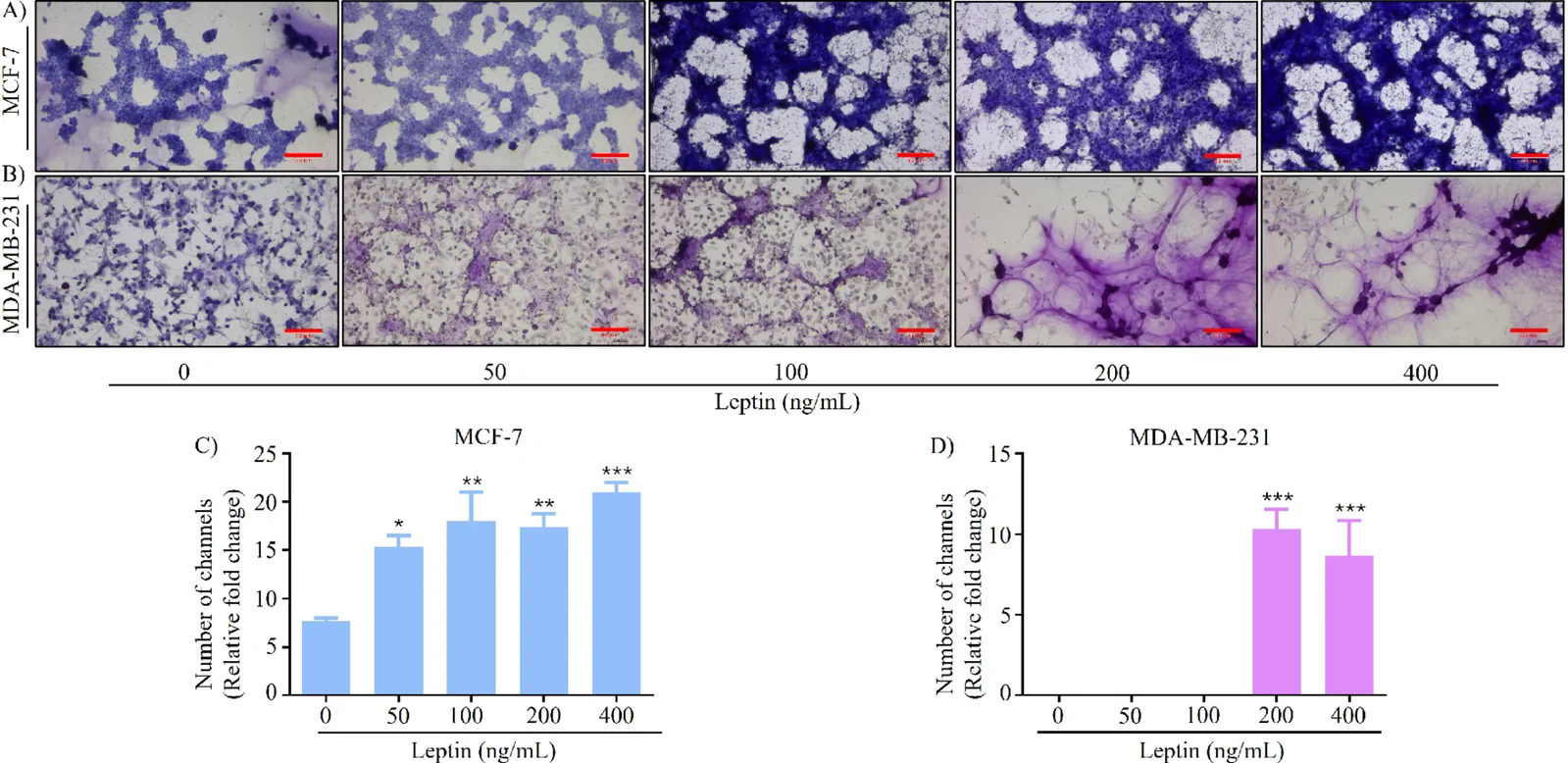
Leptin induces VM in MCF-7 and MDA-MB-231 cells. **A**–**B**. Representative PAS-stained images showing tubular channel formation following leptin treatment in MCF-7 and MDA-MB-231 cells, magnification: 10×. Pink-violet coloration indicates carbohydrate content. C–D) Quantification of channel formation in MCF-7 and MDA-MB-231 cells. Data are presented as mean ± SEM. One-way ANOVA and Dunnett’s test: *p < 0.05, ** *p* < 0.01, *** *p* < 0.001. Scale bar = 0.2 mm

PAS staining revealed extracellular matrix patterns associated with VM. MCF-7 cells showed a dose-dependent increase in the formation of defined tubular channels surrounding cell-free spaces, consistent with tubular-type VM, with detectable effects at 50 ng/mL (15.33 ± 1.20, *p* < 0.0283) and reaching a maximum at 400 ng/mL (21.00 ± 1.00 vs. 7.67 ± 0.33, *p* < 0.0007, Fig. 3A and C).

In contrast, MDA-MB-231 cells formed extracellular matrix patterns in the form of branched loops, with cells integrating into the three-dimensional extracellular matrix networks, consistent with matrix-type VM. This process began at 50 ng/mL of leptin and showed a defined formation at 200 (10.33 ± 1.20, *p* < 0.0002) and 400 ng/mL (8.67 ± 2.19 vs. 0.00 ± 0.00, *p* < 0.0009, *n* = 3 independent experiments, Fig. 3B and D).

Interestingly, our observations suggest that leptin stimulation may promote the formation of structures consistent with either tubular-type or matrix-type VM, depending on the cellular subtype. However, since the identification of these structures is based mainly on morphological characteristics and PAS staining, future studies should focus on confirming the presence of functional lumens and perfusable structures to rule out alternative explanations such as increased extracellular matrix deposition or simple cellular alignment.

### Leptin-induced angiogenesis is mediated by FAK signaling

**Fig. 4:**
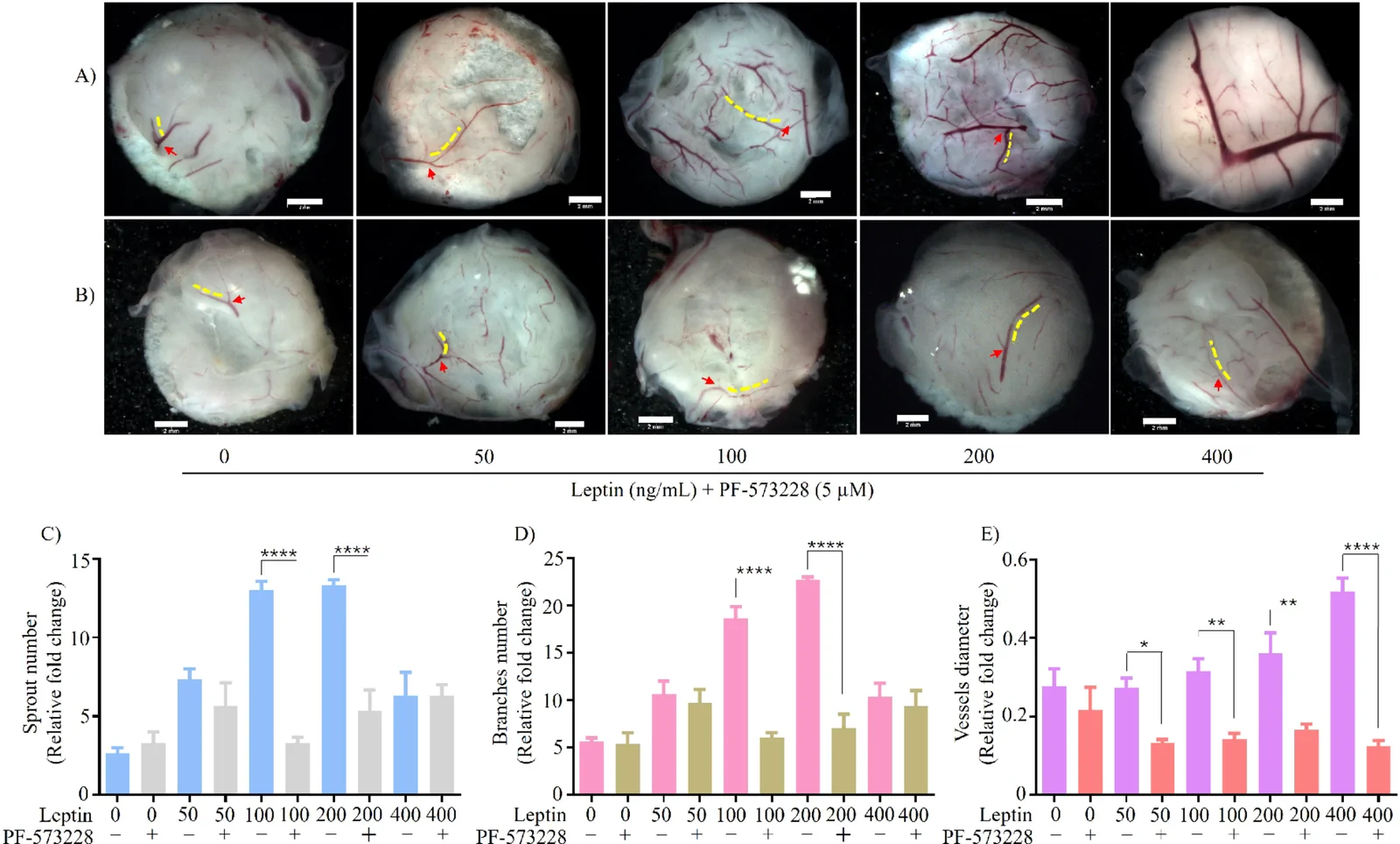
Leptin induces angiogenesis via FAK signaling. (**A**) Representative images of blood vessel development after leptin treatment. (**B**) Vessel development following leptin stimulation in the presence of PF-573,228 (5 µM). (**C**) Quantification of capillary sprouting. (**D**) Vessel branching analysis. (**E**) Capillary diameter measurements. Red arrows indicate capillary sprouts; yellow dashed lines indicate vessel branching. In the graphs, blue, pink, and purple represent leptin-only treatments, whereas gray, brown, and orange represent leptin + PF-573,228. Data are presented as mean ± SEM. One-way ANOVA and Newman–Keuls test: *p < 0.05, ** *p* < 0.01, *** *p* < 0.001, **** *p* < 0.0001. Scale bar = 2 mm

FAK is a key regulator of leptin-mediated processes, including cell migration, MMP secretion, and invasion, contributing to a more aggressive breast cancer phenotype [19]. Therefore, we investigated the role of FAK in leptin-induced angiogenesis. To this end, we performed CAM assays using the FAK inhibitor PF-573,228 (5 µM). FAK inhibition reduced both sprouting and branching at 100 (3.33 ± 0.33 vs. 13.00 ± 0.58 and 6.00 ± 0.58 vs. 18.67 ± 1.20, respectively, *p* < 0.0001) and 200 ng/mL of leptin (5.33 ± 1.33 vs. 13.33 ± 0.33 and 7.00 ± 1.53 vs. 22.67 ± 0.33, respectively, *p* < 0.0001, *n* = 3, Fig. 4A–D). Additionally, FAK inhibition significantly decreased capillary diameter at all tested leptin concentrations (0.13 ± 0.00 vs. 0.27 ± 0.02, *p* < 0.05; 0.14 ± 0.01 vs. 0.32 ± 0.03, *p* < 0.01; 0.17 ± 0.02 vs. 0.36 ± 0.01, *p* < 0.01 and 0.12 ± 0.01 vs. 0.52 ± 0.03 *p* < 0.0001 respectively, *n* = 3 (9 measurements), Fig. 4E). These results suggest that leptin acts as an angiogenic factor through FAK and that this kinase also appears to regulate vascular remodeling in both leptin-dependent and leptin-independent manners.

### Leptin induces vasculogenic mimicry via FAK signaling

**Fig. 5:**
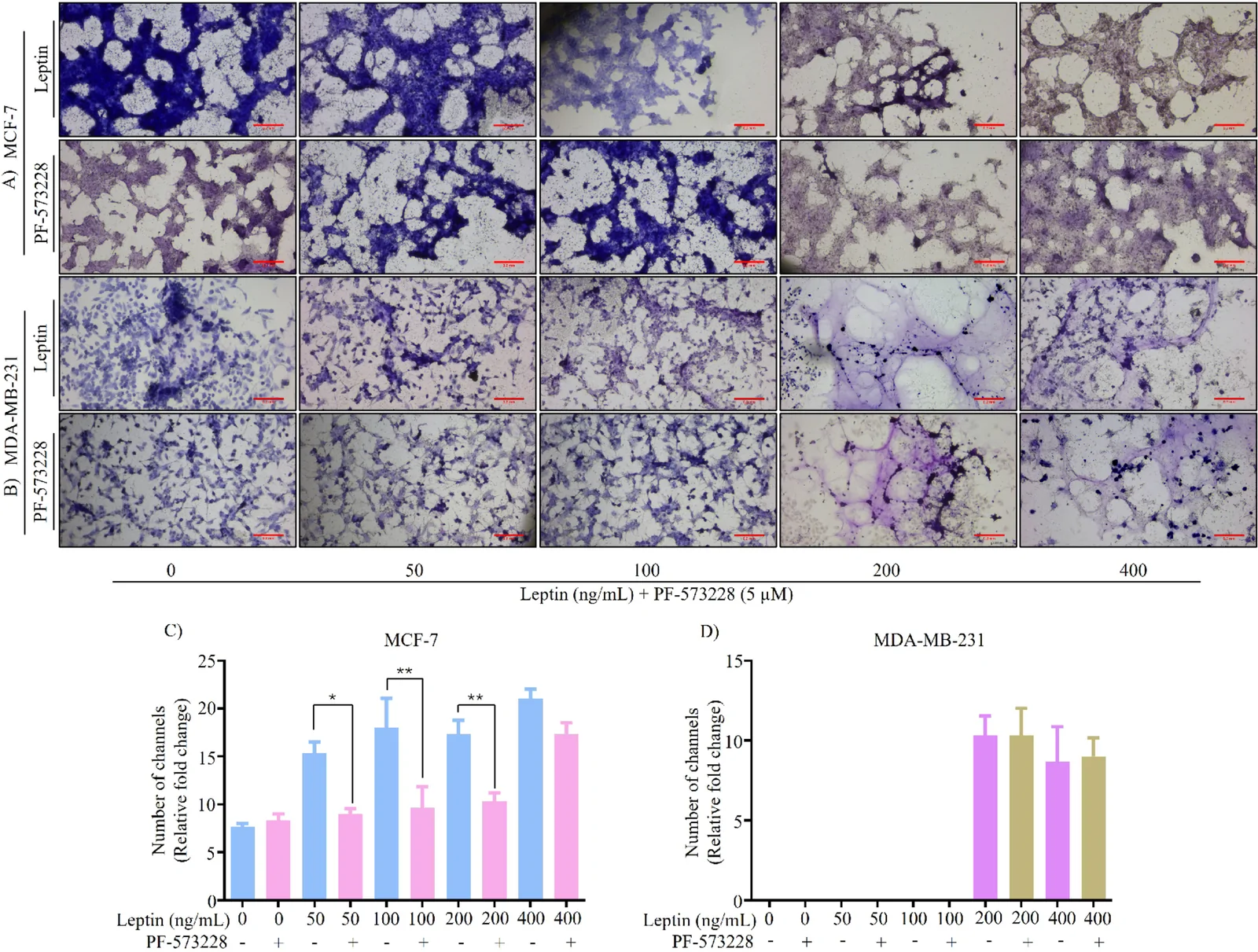
Leptin induces VM through FAK in breast cancer cells. **A**–**B.** Channel formation in MCF-7 and MDA-MB-231 cells treated with leptin ± PF-573,228 for 48 h (10× objective). Pink-violet staining indicates carbohydrate content. **C**–**D.** Quantification of channel formation in MCF-7 and MDA-MB-231 cells. In the graphs, blue and purple represent leptin-only treatments, while pink and brown represent leptin + PF-573,228. Data are presented as mean ± SEM. One-way ANOVA and Newman–Keuls test: *p < 0.05, ** *p* < 0.01, *** *p* < 0.001. Scale bar = 0.2 mm

Leptin promotes VM formation in breast cancer cells; however, the pathways and molecules regulated by this adipokine during this process have been poorly investigated. To evaluate the involvement of FAK in leptin-induced VM, MCF-7 and MDA-MB-231 cells were treated with leptin and PF-573,228 (5 µM) for 48 h. FAK inhibition significantly reduced leptin-induced channel formation at concentrations of 50 (9.00 ± 0.58 vs. 15.33 ± 1.20, *p* < 0.05), 100 (9.67 ± 2.19 vs. 18.00 ± 3.05, *p* < 0.01), and 200 (10.33 ± 0.88 vs. 17.33 ± 1.45, *p* < 0.01, *n* = 3) ng/mL in MCF-7 cells (Fig. 5A and C), indicating that this process is FAK-dependent in the luminal model. In contrast, in triple-negative cells, the formation of matrix-type structures was unaffected by FAK inhibition (Fig. 5B and D), suggesting that the effect of leptin is independent of this signaling pathway. Collectively, these results demonstrate a differential dependence on FAK between luminal and triple-negative breast cancer subtypes.

### Leptin promotes the expression of angiogenesis- and vasculogenic mimicry-related proteins via FAK signaling

**Fig. 6:**
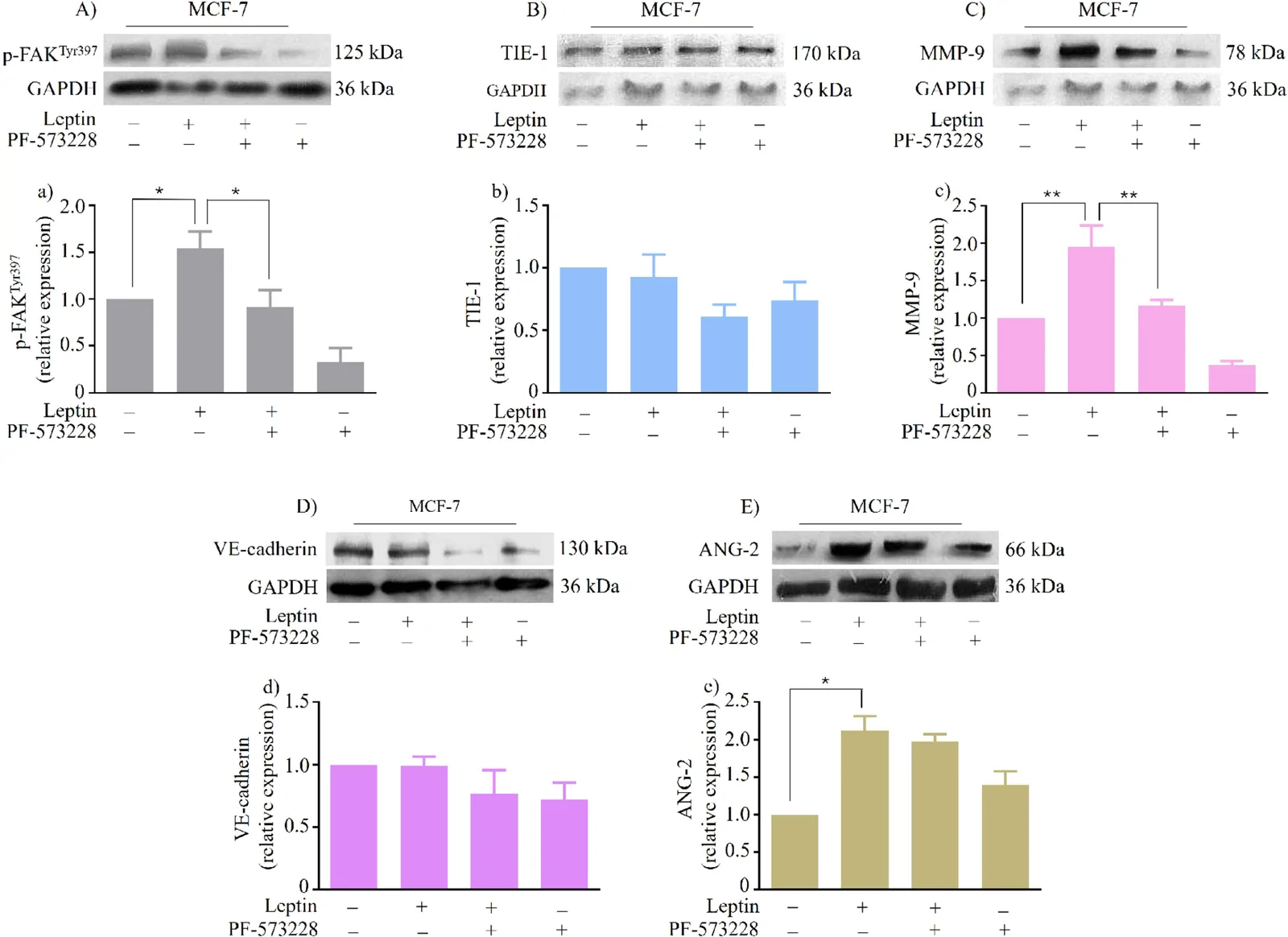
Leptin increases the expression of angiogenesis- and VM-related proteins via FAK signaling in MCF-7 cells. Representative western blots of (**A**) p-FAK^Tyr397^, (**B**) TIE-1, (**C**) MMP-9, (**D**) VE-cadherin, (**E**) ANG-2 in MCF-7 cells treated with leptin (200 ng/mL), with or without PF-573,228 (5 µM). Densitometric analyses of (a) p-FAK^Tyr397^, (b) TIE-1, (c) MMP-9, (d) VE-cadherin, (e) ANG-2. Data are presented as mean ± SEM. Statistical significance was determined using the Newman–Keuls test: *p < 0.05, ** *p* < 0.01, *** *p* < 0.001

To elucidate the molecular mechanisms underlying leptin-induced angiogenesis and VM-mediated FAK signaling, we performed western blot analyses of lysates from MCF-7 and MDA-MB-231 cells treated with leptin, in the presence or absence of the FAK inhibitor PF-573,228. Leptin stimulation enhanced FAK phosphorylation at Tyr397 (1.54 ± 0.18 vs. 1.00 ± 0.00, *p* < 0.05, *n* = 3, Fig. 6A-a) and increased MMP-9 expression (1.95 ± 0.28 vs. 1.00 ± 0.00, *p* < 0.01, *n* = 3, Fig. 6C-c) in MCF-7 cells. Leptin also increased ANG-2 levels in a FAK-independent manner (2.12 ± 0.19 vs. 1.00 ± 0.00, *p* < 0.05, *n* = 2, Fig. 6E-e), without observable changes in TIE-1 (0.92 ± 0.18 vs. 1.00 ± 0.00, *n* = 3, Fig. 6B-b) and VE-cadherin (0.99 ± 0.07 vs. 1.00 ± 0.00, *n* = 3, Fig. 6D-d) expression following leptin treatment. These molecular changes are associated with the formation of tubular VM structures and the vascular remodeling previously described in this model.

**Fig. 7:**
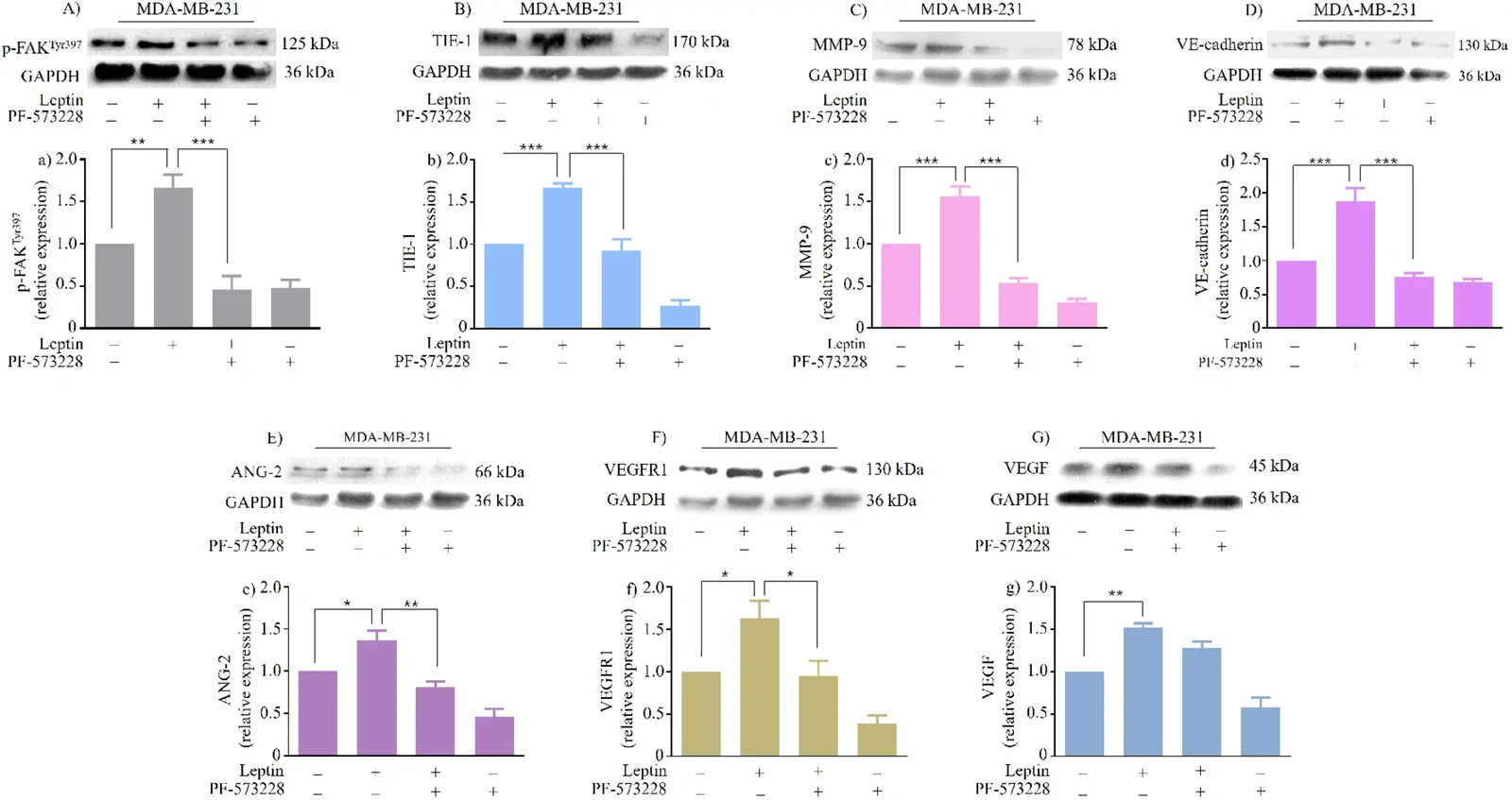
Leptin increases the expression of angiogenesis- and VM-related proteins via FAK signaling in MDA-MB-231 cells. Representative western blots of (**A**) p-FAK, (**B**) TIE-1, (**C**) MMP-9, (**D**) VE-cadherin, (**E**) ANG-2, (**F**) VEGFR1, (**G**) VEGF in MDA-MB-231 cells treated with leptin (100 ng/mL), with or without PF-573,228 (5 µM). Densitometric analyses of (a) p-FAK, (b) TIE-1, (c) MMP-9, (d) VE-cadherin, (e) ANG-2, (f) VEGFR1, (g) VEGF. Data are presented as mean ± SEM. Statistical significance was determined using the Newman–Keuls test: *p < 0.05, ** *p* < 0.01, *** *p* < 0.001

In MDA-MB-231 cells, leptin also promoted FAK phosphorylation at Tyr397 (1.67 ± 0.15 vs. 1.00 ± 0.00, *p* < 0.01, *n* = 3, Fig. 7A-a), which was associated with increased expression of TIE-1 (1.67 ± 0.05 vs. 1.00 ± 0.00, *p* < 0.001, *n* = 3, Fig. 7B-b), MMP-9 (1.56 ± 0.12 vs. 1.00 ± 0.00, *p* < 0.001, *n* = 3, Fig. 7C-c), VE-cadherin (1.88 ± 0.20 vs. 1.00 ± 0.00, *p* < 0.001, *n* = 3, Fig. 7D-d), ANG-2 (1.36 ± 0.11 vs. 1.00 ± 0.00, *p* < 0.05, *n* = 3, Fig. 7E-e), and VEGFR1 (1.63 ± 0.20 vs. 1.00 ± 0.00, *p* < 0.05, *n* = 3, Fig. 7F-f). The FAK-dependent overexpression of these proteins could contribute to the formation of networks characteristic of matrix-type VM and to the tumor angiogenesis observed in this aggressive breast cancer subtype. Notably, leptin-induced VEGF expression was independent of FAK signaling (1.51 ± 0.05 vs. 1.00 ± 0.00, *p* < 0.01, *n* = 3, Fig. 7G-g), suggesting a distinct regulatory mechanism in MDA-MB-231 cells.

## Discussion

Leptin is a multifunctional adipokine closely associated with breast cancer progression and aggressiveness. Leptin regulates multiple biological processes, including cell proliferation, adhesion, invasion, migration, inflammation, and angiogenesis [28]. Among the signaling pathways mediating leptin effects is FAK, which plays a fundamental role in the development of invasive phenotypes and in the regulation of cell migration and invasion [19, 20]. However, the role of FAK during leptin-induced tumor angiogenesis is not yet fully understood.

Accordingly, the present study aimed to evaluate leptin-induced angiogenesis and observed significant increases in vascular sprouting and branching, as well as in vessel diameter, suggesting vascular remodeling. Notably, these effects were attenuated following FAK inhibition, indicating that this signaling pathway plays a key role in leptin-mediated angiogenesis.

Although the leptin–FAK axis has not been widely studied in the context of angiogenesis, there is evidence demonstrating the proangiogenic effect of leptin in endothelial and tumor models, including breast cancer [29], glioblastoma multiforme [30], cholangiocarcinoma [31], melanoma [32], and chondrosarcoma [33]. Therefore, these data suggest that FAK activation by leptin likely promotes vascular remodeling by stimulating endothelial cell proliferation and migration, as well as cytoskeletal organization and extracellular matrix remodeling through MMP-2 and MMP-9 [34–36]. Additionally, this effect may be mediated through the regulation of key angiogenic factors such as VEGF, VEGFR1, and VEGFR2, as previously described in other angiogenic contexts [35, 37]. The increase in vascular lumen could be influenced by leptin-induced expression of interleukin-1, endothelial nitric oxide synthase, cyclooxygenase-2, and prostacyclin, which are well-established regulators of vascular tone and endothelial function [38–40].

In this context, our data showed significant differences between the two cellular models used. MCF-7 tumors showed decreased vascular density and increased vessel diameter after leptin treatment, suggesting vascular remodeling. This process may reflect an energy conservation strategy under conditions of limited angiogenic signaling [41, 42], allowing the maintenance of tumor perfusion and favoring metastasis [43]; through structural modifications of pre-existing vessels, rather than extensive formation of new vasculature.

In contrast, MDA-MB-231 tumors showed an increase in both vascular density and vessel caliber after leptin treatment. These differences may reflect the intrinsic angiogenic potential of each molecular subtype. Gene expression studies have shown that triple-negative cells express higher levels of proangiogenic molecules (e.g., VEGF, MMP, ANG), which are associated with greater invasiveness [44]. In contrast, luminal A cells exhibit lower angiogenic capacity and tumorigenicity. Since their growth and proliferation depend on estrogen, they show low intrinsic expression of proangiogenic factors, limiting blood vessel formation and tumor growth [44, 45].

In agreement with our molecular analysis, in which leptin/FAK signaling increased the expression of multiple proangiogenic proteins in MDA-MB-231 cells, including TIE-1, ANG-2, MMP-9, VE-cadherin, and VEGFR1, and increased VEGF independently of this pathway. Whereas in MCF-7 cells, FAK-dependent expression of MMP-9 and FAK-independent expression of ANG-2 were observed. Together, these molecules play key roles in tumor angiogenesis: MMP-9 facilitates extracellular matrix remodeling and the release of angiogenic factors, while ANG-2 contributes to vascular remodeling and increased vessel diameter. Meanwhile, VE-cadherin and TIE-1 are involved in cell adhesion, vascular integrity, and vascular maturation. In contrast, the VEGF/VEGFR1 axis regulates endothelial cell proliferation, migration, and activation, as well as vascular sprouting, permeability, and vasodilation [6, 46–54].

These mechanisms could explain the highly angiogenic and aggressive phenotype characteristic of the triple-negative subtype. Likewise, in MCF-7 tumors, the involvement of MMP-9 and ANG-2 suggests their role as key regulators of leptin-induced vascular plasticity, contributing to different mechanisms of vascular adaptation depending on the molecular subtype.

Additionally, this study showed increased expression of VEGF and N-cadherin in both tumor models following leptin treatment, which is consistent with previous reports linking the overexpression of these proteins to treatment resistance, MMP-9 secretion, and metastasis to organs such as the liver, pancreas, lungs, and lymph nodes [55–58].

In addition to its angiogenic effects, leptin also promotes VM formation. In our MDA-MB-231 cell model, leptin induced VM-associated matrix-type formations independently of FAK signaling.

Matrix-type VM has been associated with poor prognosis, increased diffusion of oxygen and nutrients, and aggressive tumor phenotypes [59, 60]. Although it has been demonstrated that leptin induces VM in MDA-MB-231 cells via molecules such as aquaporin-1, VE-cadherin, Twist, and laminin γ5 [61], the mechanisms driving matrix-type VM remain poorly understood. In our study, we observed the expression of key proteins associated with VM formation, including MMP-9, VE-cadherin, VEGF, and VEGFR1. These molecules participate in processes such as extracellular matrix plasticity, cell–cell adhesion, and regulation of pathways related to matrix remodeling [14, 62–65]. However, despite several of these proteins being regulated by FAK signaling, inhibition of this kinase did not affect matrix-type VM formation in MDA-MB-231 cells. This finding suggests that although these proteins are present, their participation in this process may depend on alternative mechanisms that are independent of FAK. In this regard, it is possible that leptin also regulates PAS-positive extracellular matrix components, such as laminin, heparan sulfate proteoglycans, collagen IV, and collagen VI, which are key elements in matrix-type VM formation [40, 66].

Interestingly, VEGF expression was upregulated independently of FAK, suggesting the involvement of alternative signaling pathways. This could be explained by activation of previously described pathways for leptin-induced VEGF regulation, such as JAK2/STAT3, MAPK (p38, ERK, and JNK), NF-κB, HIF-1α, IL-1, and AP-1 [33, 67]. Together, these results highlight the complexity of the mechanisms involved and the need for further studies to clarify the functional role of these molecules in matrix-type VM.

In contrast to observations in MDA-MB-231 cells, leptin induced tubular-type VM formation in the luminal model in a FAK-dependent manner, accompanied by MMP-9 expression. This molecule could contribute to extracellular matrix remodeling, thereby favoring the formation of channel-like structures [62]. This subtype of VM has also been associated with EMT, a process that enhances tumor cell plasticity and extracellular matrix reorganization [60]. In this context, leptin/FAK signaling could regulate tubular VM through activation of transcription factors such as Twist and metalloproteinases such as MMP-2 and MMP-9 [20]. Overall, our findings suggest that leptin is a key modulator of tumor vascular plasticity in breast cancer, contributing to tumor growth, perfusion, and invasion.

Despite the promising results, several limitations of this study should be considered. First, the CAM assay and the in vitro models used, although robust, lack the full complexity of the human tumor microenvironment, including immune components and stromal interactions. Likewise, although the CAM model enables efficient evaluation of angiogenic processes, the lack of in vivo mammalian models remains an important limitation, as it does not fully recapitulate the tumor’s physiological and systemic dynamics.

Additionally, only two breast cancer cell lines were analyzed; therefore, extrapolation to other molecular subtypes should be made with caution. Future studies using orthotopic models or patient-derived xenografts will be essential to confirm the role of leptin-FAK signaling in vivo.

Another limitation to consider is the possible variability in physiological leptin concentrations, particularly in different metabolic contexts such as obesity, which could influence the magnitude of the observed responses.

From a translational perspective, our findings support the leptin-FAK axis as a potential therapeutic target in breast cancer. Given the strong association between obesity, hyperleptinemia, and poor breast cancer prognosis, disruption of this signaling pathway could represent a promising strategy to inhibit angiogenesis and vascular metastasis, particularly in leptin-responsive tumors. In this regard, combining FAK inhibitors with currently used antiangiogenic therapies could have additive or potentially synergistic effects, improving therapeutic efficacy through complementary effects on tumor vascularization.

However, the clinical translation of these strategies may face important challenges, including tumor heterogeneity, variability in leptin levels associated with metabolic conditions such as obesity, and the potential compensatory activation of alternative signaling pathways. These aspects should be considered in future studies to optimize the clinical applicability of these therapeutic approaches.

## Conclusion

**Fig. 8:**
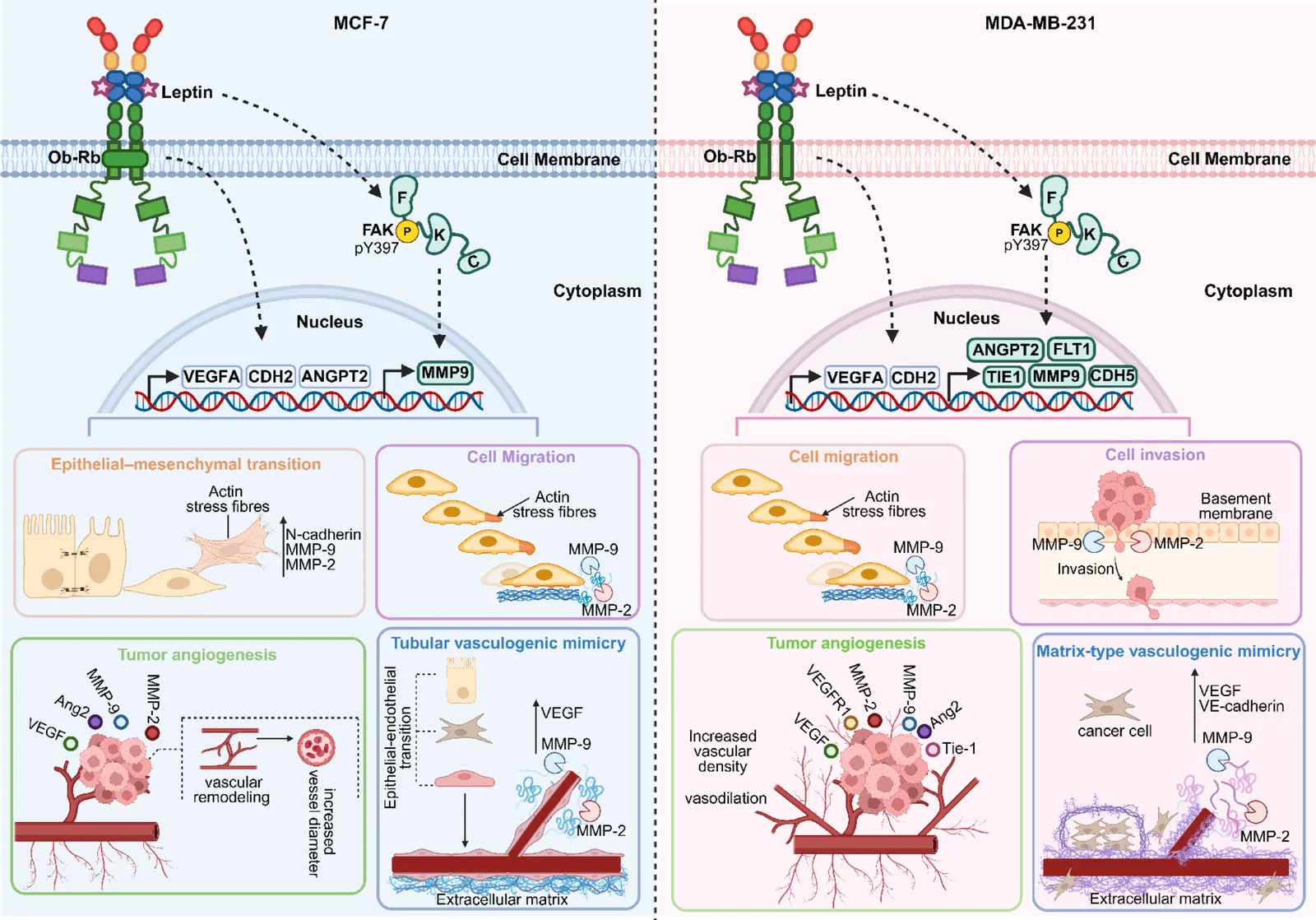
Proposed model of the regulation of leptin-induced markers and biological processes through FAK signaling in MCF7 and MDA-MB-231 cells

This study demonstrates that leptin promotes tumor vascularization in breast cancer through multiple mechanisms. Leptin regulates capillary sprouting, branching, and vasodilation via non-canonical FAK signaling, while also enhancing the expression of VEGF, N-cadherin, TIE-1, MMP-9, VE-cadherin, ANG-2, and VEGFR1. Furthermore, leptin induces tubular-type VM in MCF-7 cells via FAK and matrix-type VM in MDA-MB-231 cells independently of FAK. Collectively, these findings indicate that leptin-driven angiogenesis and VM may contribute to increased migration, invasion, and metastasis in breast cancer, highlighting FAK signaling as a potential therapeutic target (Fig. 8).

## Data Availability

All raw data, including imaging files, quantified values, and western blot scans, are available from the corresponding author upon reasonable request.
